# Surgical Treatment and Rehabilitation Strategies for Upper and Lower Extremity Lymphedema: A Comprehensive Review

**DOI:** 10.3390/medicina58070954

**Published:** 2022-07-19

**Authors:** Alessandro de Sire, Luigi Losco, Lorenzo Lippi, Davide Spadoni, Juste Kaciulyte, Gokhan Sert, Paola Ciamarra, Marco Marcasciano, Roberto Cuomo, Alberto Bolletta, Marco Invernizzi, Emanuele Cigna

**Affiliations:** 1 Physical Medicine and Rehabilitation Unit, Department of Medical and Surgical Sciences, University of Catanzaro “Magna Graecia”, 88100 Catanzaro, Italy; alessandro.desire@unicz.it; 2 Plastic Surgery and Microsurgery Unit, Department of Translational Research and New Technologies in Medicine and Surgery, University of Pisa, 56122 Pisa, Italy; luigi.losco@gmail.com (L.L.); emanuele.cigna@unipi.it (E.C.); 3 Department of Medicine, Surgery and Dentistry, University of Salerno, 84081 Salerno, Italy; 4 Physical and Rehabilitative Medicine, Department of Health Sciences, University of Eastern Piedmont, 28100 Novara, Italy; lorenzolippi.mt@gmail.com (L.L.); marco.invernizzi@med.uniupo.it (M.I.); 5 Translational Medicine, Dipartimento Attività Integrate Ricerca e Innovazione (DAIRI), Azienda Ospedaliera SS. Antonio e Biagio e Cesare Arrigo, 15121 Alessandria, Italy; 6 Department of Plastic and Reconstructive Surgery, China Medical University Hospital, China Medical University, Taichung 40447, Taiwan; davidespadoni@hotmail.it (D.S.); justekc@gmail.com (J.K.); drgokhansert@gmail.com (G.S.); 7 Department of Experimental Medicine, University of Campania “Luigi Vanvitelli”, 80138 Naples, Italy; paolaciamarra8@gmail.com; 8 Plastic and Reconstructive Surgery Unit, Department of Experimental and Clinical Medicine, University of Catanzaro “Magna Graecia”, 88100 Catanzaro, Italy; m.marcasciano@unicz.it; 9 Unit of Plastic and Reconstructive Surgery, Department of Medicine, Surgery and Neuroscience, University of Siena, 53100 Siena, Italy; roberto.cuomo@unisi.it

**Keywords:** lymphedema, breast cancer-related lymphedema, plastic surgery, rehabilitation, manual lymphatic drainage, vascularized lymph node transfer, lymphaticovenous anastomosis, Charles procedure, lymphatic surgery

## Abstract

Lymphedema is a chronic disabling condition affecting a growing number of patients worldwide. Although lymphedema is not life-threatening, several reports underlined detrimental consequences in terms of distress, pain, functional impairment, and infections with a relevant decrease in quality of life. Currently, there is no cure, and the therapeutic management of this condition aims at slowing down the disease progression and preventing secondary complications. Early diagnosis is paramount to enhance the effects of rehabilitation or surgical treatments. On the other hand, a multidisciplinary treatment should be truly integrated, the combination of microsurgical and reductive procedures should be considered a valid strategy to manage extremity lymphedema, and rehabilitation should be considered the cornerstone of the multidisciplinary treatment not only for patients not suitable for surgical interventions but also before and after surgical procedures. Therefore, a specialized management of Plastic Reconstructive Surgeons and Physical and Rehabilitative Medicine physicians should be mandatory to address patients’ needs and optimize the treatment of this disabling and detrimental condition. Therefore, the aim of this review was to characterize the comprehensive management of lymphedema, providing a broad overview of the potential therapy available in the current literature to optimize the comprehensive management of lymphedema and minimize complications.

## 1. Introduction

The word “lymphedema” describes a progressive and chronic collection of protein-rich lymphatic fluid in the interstitial space due to lymph nodes and/or lymphatic vessels impairment; overall, 140–250 million people are estimated to be affected by lymphedema worldwide [1,2]. The onset is subtle; a slight tightness of clothes and a feeling of heaviness could be complained in the concerned limb. Gradually, edema becomes apparent and evolves from pitting to non-pitting and, later, to soft tissue proliferation and fibrosis that impairs, even more, the activity of the lymphatic system [3]. Lymphedema is usually not life-threatening, but due to the swelling of the affected limb, distress, pain, loss of function, and frequent infections may occur. Moreover, it has a very deleterious effect on self-esteem and causes impairments in body perception [4,5,6]. Primary lymphedema is the consequence of a progressive swelling without connection to any medical condition; it is caused by unusual development of the lymphatic system, and the onset could range from childhood to adult age [7]. In secondary cases, direct trauma, infection, surgery for cancer, or radiotherapy could be responsible for the impairment of the lymphatic system. To date, the increase in overall survival of cancer patients has been associated with a progressive increase in long-term complications, including lymphedema, that represents a critical issue in the current health care system [8,9]. Currently, there is no chance to cure this condition, and, regardless of the etiology, the therapeutic aim is to slow down the advancement of the disease and prevent secondary complications; early diagnosis is paramount to establish an effective physical therapy protocol or surgical treatment after appropriate patient information [10,11,12].

In this scenario, international guidelines defined a conservative multitarget comprehensive approach commonly defined as Complex Decongestive Therapy (CDT). It includes manual lymph drainage (MLD), skin care, specialized exercises, compression garments and self-education [13]. CDT is widely accepted as the universal first-line therapy for extremity lymphedema, and surgical procedures are commonly considered when conservative treatments are no longer effective.

The debulking surgeries aim to remove the affected skin and subcutaneous tissue that display final adipose and fibrotic changes. Suction-assisted lipectomy (SAL) is performed for initial adipose tissue accumulation, while in case of severe fibrosis, direct excision becomes necessary, and a Radical Reduction with Preservation of Perforators (RRPP) or modified Charles’ procedure should be carried out.

**Figure 1:**
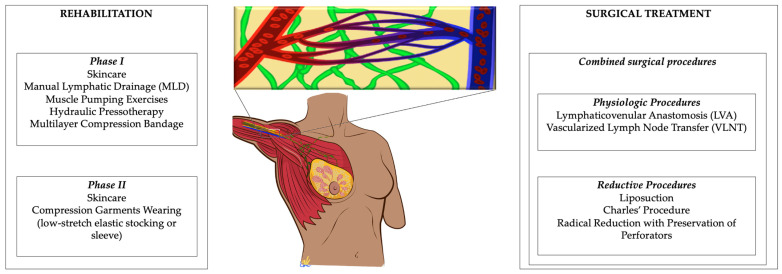
Management of lymphedema from a Plastic Reconstructive Surgeons and Physical and Rehabilitative Medicine point of view.

On the other hand, a wider understanding of lymphedema pathophysiology and novel advances in microsurgery introduced new physiologic procedures that aim to restore the impaired lymphatic flow, such as Lymphaticovenular Anastomosis (LVA) and Vascularized Lymph Node Transfer (VLNT) (Figure 1).

However, despite the multitude of surgical treatment options, there is currently no agreement on the indications, timing/staging and potential combination of these procedures [14,15]. Indeed, we have to highlight a lack of clear evidence about the effectiveness of physiologic procedures for the treatment of lymphedema. Intrinsic features of lymphedema itself with its various pathophysiology and presentations, heterogeneity in lymphedema staging and a lack of consensus among the experts regarding the most appropriate protocol for lymphedema treatment are major flaws in the path of building an evidence-based treatment protocol.

Thus, by the present comprehensive review, we aimed to provide a broad overview of the comprehensive management of lymphedema in the current literature from a Plastic Reconstructive Surgeons and Physical and Rehabilitative Medicine point of view.

## 2. Research Methodology

Scientific literature research has been performed on PubMed/Medline, Scopus, Cochrane Central Register of Controlled Trials (CENTRAL), Physiotherapy Evidence Database (PEDro), and Web of Science (WoS) using the following Mesh terms: “Lymphedema”, “Breast Cancer Lymphedema”, “Rehabilitation”, “Physical Therapy”, “Exercise”, “Manual Therapy”, “Manual lymphatic drainage”, “Liposuction”, “Anastomosis”, “Vascularized Lymph Node Transfer”, and “Lymphatic surgery”, “Lymphaticovenular Anastomosis”, “Charles Procedure”.

The literature research was performed between October 2021 and March 2022 by two independent reviewers. Subsequently, two reviewers independently screened the studies for eligibility. If consensus was not reached, a third reviewer was asked.

We considered only studies assessing adult patients (aged > 18 years) with lymphedema or at high risk of lymphedema. We excluded all the studies in languages other than English, studies without full text available, studies involving animals, conference abstracts, masters, or doctorate theses.

A qualitative method has been used for the data extraction and the data synthesis. More in detail, lymphedema staging, conservative management, and surgical management of lymphedema were extracted and synthesized in the manuscript.

Both the data extraction and the data synthesis have been independently performed by two reviewers. In case of disagreement, if consensus was not reached by discussion, a third reviewer was asked.

## 3. Lymphedema Staging

Lymphedema staging systems are important tools in the management of the disease; they are an effective aid to set an effective protocol. The International Society of Lymphology has established a staging system for lymphedema [16], and it is the most widely utilized staging system for identifying the progression and/or severity of disease. It classifies lymphedema into three clinical stages: patients are categorized as Stage 0 (latent or sub-clinical lymphedema) when lymph transport is impaired but swelling or edema is not measurable. Stage I (spontaneously reversible lymphedema) is assessed with measurable swelling and pitting of the skin due to accumulation of lymph; however, it decreases with limb elevation or compression garments. In Stage II (spontaneously irreversible lymphedema), significant adipose tissue deposition and protein-rich fluid accumulation prevent limb elevation or compression garments from being an effective approach to downsize symptoms. In late Stage II, the limb may display increase in subcutaneous fat tissue and fibrotic changes. Finally, Stage III (lymphostatic elephantiasis) is the most severe stage of lymphedema. It is characterized by severe swelling, extensive deposition of fat and fibrosis and significant skin thickening.

Further classifications combining both physical (phenotypic) findings with functional lymphatic imaging (either ICG Lymphography and Lymphoscintigraphy) [17,18] as well as immunohistochemical changes determined by biopsy of nodes/vessels have been proposed [19]. The advancement of the disease process will indicate options available for treatment.

## 4. Management Strategies for Lymphedema

It is widely recognized that the early detection and precise diagnosis of lymphedema play a key role in lymphedema management, allowing early specific treatments. Particular attention should be paid to the early detection of limb volume increasing in order to manage lymphedema in lower stages to avoid complications and disease progression [20].

### 4.1. Conservative Therapy

In accordance with the most recent guidelines [16], CDT should include two phases:

1. Phase I: characterized by skincare, manual lymphatic drainage (MLD), with or without deeper techniques including muscle pumping exercises or hydraulic pressotherapy, followed by multilayer compression bandage, aiming at improving lymphedema volume.
2. Phase II: characterized by skincare and compression garments wearing, including low-stretch elastic stocking or sleeve, aiming at avoiding complications and conserving the results obtained in Phase I.

In particular, as a matter of fact, compression should be considered the cornerstone of the CDT. However, there is large heterogeneity in terms of the methods, times, and duration of this treatment [16]. Recently, the randomized, single-blind, clinical trial by Torres-Lacomba et al. [21] compared the effects of four types of bandages and kinesio-tape in terms of lymphedema volume reduction in patients suffering from ULL. The authors reported significant differences in terms of lymphedema volume after three weeks of treatment, with the larger improvement in the simplified multilayer group (59.5%), which was treated with a compression composed of two layers combining inelastic and elastic bandages. In contrast, Kinesio-tape was the most comfortable compression strategy, while the multilayer bandage was the most uncomfortable (*p* < 0.001). Similarly, in 2021, our research group introduced an instantly adjustable inelastic compression device in the CDT of lymphedema. Our results showed BCRL patients had a significant reduction in lymphedema volume after the treatment (*p* = 0.001) without reporting significant side effects. These findings suggested the potential implications of this instantly adjustable inelastic compression device in the rehabilitative management of BCRL patients [13].

Accordingly, the RCT by Tantawy et al. [22] compared the effects of Kinesio-tape and compression garments in patients suffering from BCRL. In this study, significant differences were found between the Kinesio-tape group and the compression garment group in terms of lymphedema volume, upper limb function, muscle strength, and quality of life. In addition, only the Kinesio-tape group have a significant improvement in upper limb function and muscle strength (*p* < 0.05).

Concurrently, the intensity of CDT is a critical issue in the current literature, with a high heterogeneity of intervention protocols. Recently, a high-intensity study protocol has been proposed by Pereira de Godoy et al. [23] in a large trial involving over 400 participants affected by LLL. The author reported a significant improvement of stages II and III lymphedemas in terms of limb volume after the Godoy method, consisting of eight hours/day of mechanical lymphatic drainage combined with 15 min/day of cervical stimulation, which was followed by compression stockings alternated with multilayer bandages. To date, compression is considered the most important component of the CDT [24]; however, several compression strategies and devices that are currently available severely influence the standardization of lymphedema intervention in clinical trials [25].

#### 4.1.1. Instrumental Physical Therapy

In the complementary management of ULL, instrumental physical therapy has been proposed to have a role in volume reduction in lymphedema and chronic inflammation regulation [26,27]. In particular, the recent study by Lee et al. [28] assessed the effects of extracorporeal shockwave therapy (ESWT) in the most fibrotic lesions, two times a week for 3 weeks, which was combined with CDT in patients suffering from BCRL. The authors reported significant improvement in lymphedema and significant differences between the control group (CDT only) in terms of volume, the ratio of extracellular water to total body water, and skin thickness. Similarly, low-level laser therapy has been proposed to have anti-inflammatory and lymphangiogenesis effects, suggesting promising implications in lymphedema management [26]. On the other hand, the recent RCT by Kilmartin et al. [29] assessed the effect of CDT with low-level laser therapy or with sham low-level laser therapy. Despite intriguing results in terms of self-reported outcomes, there was no significant reduction in limb volume.

Intriguingly, promising results were reported in the study by Zaleska et al. [25] that assessed the effects of intermittent pneumatic compression (100–120 mmHg) in patients suffering from LLL over a period of 3 years. The authors reported significant improvements in limb circumferences and tissue tonicity assessed by tonometer without complications.

In addition to this evidence, the optimal pressure prescription of intermittent pneumatic pressure is still debated. In particular, a review assessing the effects of intermittent pneumatic pressure in both ULL and LLL underlined that high-compression pressure might be questionable, suggesting that a low pressure (ranging between 40 and 60 mmHg) might provide higher efficacy over time [30].

#### 4.1.2. Physical Activity

In contrast, strong evidence supports exercise treatment in cancer patients, with a large consensus that recommends physical activity in cancer symptom management and positive effects in overall survival [31,32]. In this scenario, the recent study by Ryans et al. [33] emphasized the need for a rehabilitative comprehensive intervention in cancer patients including physical activity not only for the positive effect in lymphedema but also aiming at improving the HR-QoL of cancer survivors, given the positive effects in physical performance, physical function, depression symptoms, sleep disturbance, fatigue, and most of the disabling symptoms commonly reported by cancer survivors [34,35].

Although exercise therapy is widely recognized to have a role in ULL management, a large number of barriers still limit physical activity in these specific patients.

In particular, the recent study by Yildiz Kabak et al. [36] reported that patients with BCRL might self-report as the most important barrier to physical exercise fatigue (64.5%), having other responsibilities (60.4%) and weather-related factors (56.2%).

Due to the functional limitations commonly associated with LLL, exercise therapy is one of the treatments proposed in lymphedema management, which is commonly combined with compression therapy [37]. In this scenario, the recent study by Abe et al. [38] assessed the effects of compression bandages and 15 min of physical exercise in volume reduction, symptoms, and tissue stiffness. The short period of physical exercise was set, aiming at improving patients’ adherence to exercise intervention, which is to date still considered one of the main limitations to treatment efficacy. The authors reported significant changes in limb volume after supine bicycle ergometer intervention, while a significant correlation between skin stiffness and the magnitude of volume reduction was found.

However, albeit there were reported promising results, there is no consensus in terms of exercise intervention in lymphedema patients [39]. The gap of knowledge in this field is probably related to the optimization of the posology of exercise prescription, which should be tailored to patients’ characteristics based on evidence-based indications. However, given a large amount of literature underlining the positive effects of physical exercise in cancer patients, physical activity in lymphedema patients should not be discouraged but strongly considered in the comprehensive management of cancer-related lymphedema [31,32,40].

#### 4.1.3. Self-Management

Nevertheless, regrettably, given the large increase in BC survivors prevalence, the cost-effectiveness and sustainability of lymphedema treatment have been largely discussed [41]. Therefore, in recent years, there is growing attention on effective strategies of self-management education for lymphedema. In this scenario, the recent study by Omidi et al. [42] assessed the effect of self-management education support in terms of fear of cancer recurrence. Although no significant effects were reported, an interesting positive trend has been found in the self-management education group.

Similarly, the recent single-blind RCT by Ligabue et al. [43] assessed the effect of self-administered CDT in patients suffering from BCRL. The treatment was taught by an expert physiotherapist during group sessions in the intervention group, whereas the control group underwent standard care. Intriguingly, after 6 months of self-administered CDT, the patients have significant benefits in pain and arm asymmetry. However, despite the promising results, no significant differences were highlighted between groups, which was probably because of the small sample. Accordingly, a recent pilot RCT by Arinaga et al. [44] reported no significant differences between standard care and 10 min holistic daily self-treatment composed of skincare, central lymphatic drainage, and gentle arm exercise.

This evidence suggested the potential implications of self-treatment administration to provide potential effective intervention to manage this disabling condition during COVID-19 spreading, given the critical issue in lymphedema management represented by the redeployment of specialized staff and the closure of outpatients’ conventional care services [45,46]. In light of these considerations, the recent paper by Noble-Jones et al. [47] proposed a supporting intervention based on teleconsultation, image sharing, virtual staff team meetings, and education, which is in accordance with the telerehabilitation trend already proposed in several reports aiming at improving rehabilitation during COVID emergency [48,49,50]. Although several therapeutic interventions require in-presence rehabilitation, telemedicine might represent a promising option for strictly monitoring lymphedema and tailoring rehabilitation to patient’s needs potentially using the technology advancements proposed in recent years [51,52].

However, it should be noted that the high heterogeneity of the studies assessing self-treatment did not allow making strong conclusions; therefore, to date, self-treatment should be considered an add-on to conventional CDT including skincare, MLD, and compression therapy.

### 4.2. Surgical Treatment

The high costs and the fact that the conservative therapy is time consuming often weaken patients’ compliance and outcomes, causing the progression of the disease [53]. Patient selection for surgery varies among institutions: for patients presenting soon after developing lymphedema, and if lymphedema is at an early stage, a complete decongestive therapy protocol for 3 to 6 months is typically established [54,55,56]; surgical intervention is indicated for those with persistent lymphedema, especially with recurrent episodes of cellulitis [54,57]. For patients presenting at an advanced stage with significant pitting edema, preoperative rehabilitation with CDT can be beneficial to optimize conditions for surgery [58]. Active cellulitis, an uncontrolled primary tumor or a local recurrence are contraindications for surgery and should undergo nonsurgical management. The surgical management of lymphedema is classified as either physiologic (reconstructive) or reductive (excisional or ablative).

#### 4.2.1. Physiologic Procedures

Physiologic methods are aimed at reducing the lymphatic burden with two main mechanisms: (1) improving lymphatic circulation by introducing healthy vascularized distant tissue comprising lymph nodes into the affected extremity (vascularized lymph node transfer, VLNT); and (2) creating shunts between the congested lymphatic ducts and the venous system proximal to the site of lymphatic obstruction (lymphaticovenular anastomosis, LVA) [59].

##### Lymphaticovenular Anastomosis (LVA)

Lymphaticovenular anastomosis (LVA) or lymphovenous bypass is a surgical procedure where an anastomosis is created between the overloaded lymphatic vessels, proximal to the site of the lymphatic obstruction, and adjacent venules. O’Brien reported encouraging results using LVA in the upper limbs as early as 1977 [60]. A growing interest in the use of this surgical method has been reported as imaging techniques (i.e., intra-operative ICG-directed lymphography), better operating microscopes, microsurgical instrumentation and sutures have become available and have improved the surgeon’s ability to find lymphatic vessels appropriate for LVA and perform supermicrosurgical anastomosis with vessels as small at 0.2 mm in diameter. Previous studies have reported that LVA seems more effective in early-stage lymphedema due to the unavailability of functional lymphatic ducts in advanced-stage lymphedema [17,61,62]. However, recent studies proposed LVA as an effective treatment also for advanced stage, and they sustained that surgical treatment for lymphedema should not be based only on the International Society of Lymphedema stage, because advanced-stage lymphedema patients with high ICG velocities can benefit from LVA alone [63,64,65].

One significant strength of the procedure is that it involves very little surgical site morbidity, as the incisions are localized to the affected limb and are 1–2 cm in length. Patients are typically discharged home on the day of surgery or after 1-day observation. In some centers, this surgery is performed under local anesthesia. A drawback of this procedure is the technical difficulty for vessels handling and anastomosis as the caliber could reach 0.2 mm; moreover, specific supermicrosurgical instruments are needed [66].

##### Vascularized Lymph Node Transfer (VLNT)

Several lymph nodes along with their vascular supply are harvested from a donor site and transferred to the affected extremity as a free tissue transfer. The presence of significant segmental dermal backflow with few or no functioning lymphatic vessels on imaging is an indication for vascularized lymph node transplantation, and its distribution may help in deciding between proximal anatomical (orthotopic) or distal non-anatomical (heterotopic) lymph node flap placement. Three recipient sites have been described for upper limb lymphedema such as the axilla, elbow, and wrist. The groin, popliteal fossa, and ankle have been described as potential recipient sites for lower extremity. The decision of recipient site is taken based on the severity of the lymphedema, recipient vessel availability, and surgeon preference [67].

To date, several studies have shown that vascularized lymph node transfer is useful in treating lymphedema. One of the larger studies by Becker et al. evaluated 1500 patients with lymphedema stage I, II, and III who had undergone vascularized lymph node transfer. The minimum follow-up was 3 years. Findings included a 98% subjective improvement. Forty percent of patients with stage I and stage II lymphedema had significant improvement and required no further conservative therapy. For patients with stage III lymphedema, 95% had some improvement and 98% remained infection free. However, the stage III patients still required conservative therapy to help control edema in the limb [68]. A meta-analysis compared the outcome of VLN transfer and LVA in extremity lymphedema [69]. The result showed that although both procedures were both efficient in a short-term outcome, patients with VLN transfer presented significant better improvement in the long term with good likelihood of discontinuing the wearing of compression garments.

The lymph node flap has a positive effect acting as an immune system organ. Lymphatic channels from the affected limb connect with the lymphatics of the lymph node flap and present antigens to the lymph nodes, which can then set an immune response and minimize the risk of infection for the affected limb. Moreover, it has been indirectly observed that most patients with recurrent bouts of cellulitis will experience a significant reduction in these episodes after the VLNT [70].

Donor sites typically exploited for lymph node harvest are lateral thoracic lymph nodes, inguinal lymph nodes, submental lymph nodes, supraclavicular lymph nodes, and gastroepiploic lymph nodes [70].

One of the major drawbacks to lymph node transfers is the potential for iatrogenic secondary lymphedema at the donor sites. One method developed to help prevent this complication is to use reverse mapping to identify and protect lymph nodes that preferentially drain the extremity and to spare these during a groin lymph node or lateral thoracic lymph node harvest. This is completed by injecting radiotracer into the subdermal plane at the web spaces of the ipsilateral limb and ICG into the subdermal plane of the ipsilateral groin or axilla. Intraoperatively, the lymph nodes identified by the ICG dye are also examined with a gamma probe. Any “hot” lymph nodes that elicit radiotracer uptake are spared and not included in the flap harvest, thus, in theory, preserving the lymph nodes, which preferentially drain the donor extremity. Dayan et al. have published very promising preliminary results using this method [71]. However, to the best of our knowledge, gastroepiploic lymph node transfer has never been associated with iatrogenic lymphedema; its main downside is the need for an abdominal surgeon for harvesting [4,72].

#### 4.2.2. Reductive Procedures

The progressive abnormal accumulation of protein-rich fluid in the interstitium triggers a cascade of adverse events culminating in fat deposition and fibrosis: Once fibrosis and lipodystrophy occur in the affected limb, correcting the fluid accumulation will not decrease the size of the limb.

In advanced-stage lymphedema, the soft tissues, which are edematous and fibrotic, those above the level of the deep fascia, are surgically removed with either direct excision or by liposuction. Excisional procedures excise the redundant subcutaneous and/or cutaneous tissue, but lymph flow is not restored [73].

##### Liposuction

Fat accumulation is one of the pathologic findings of chronic lymphedema. However, the pathophysiological mechanism of adipose tissue accumulation in lymphedema still remains controversial. Suction-assisted lipectomy has been widely utilized as a reductive method to remove the hypertrophied fat of the affected extremity. This is the less invasive method among the reductive procedures. Significant arm reduction and a reduction in cellulitis episodes have been reported after SAL [74,75], and for this reason, lymphedema-associated fat deposition should be treated with SAL primarily. However, liposuction does not provide an improvement of the lymphatic system, and therefore, patients need to wear compression garments lifelong to prevent recurrence. For this reason, for non-compliant patients, a radical reduction with a preservation of perforators (RRPP) procedure could be more efficient [76].

##### Charles’ Procedure

Charles’ procedure is the most radical among reductive procedures, and it is utilized only in the treatment of lower extremity advanced-stage lymphedema. It is the radical circumferential excision of the subcutaneous tissue and part of the fibrotic fascia of the affected limb and resurfacing with split-thickness skin grafts (STSG) [77].

The circumferential excision involves the lower limb, but the foot is excluded: only the dorsum of the foot and toes are treated, and paratenon should be preserved to sustain the skin graft. The resulting wound is then covered with split thickness skin grafts which may be harvested from the affected limb prior to excision of the tissue or from other parts of the body (if the skin of the affected limb is compromised). The disadvantages of the Charles procedure are the poor cosmetic outcome, recurrence, and the progresses of the disease on the remaining tissues. To eliminate these complications, the excisional procedure was combined with lymph node flap transfer. In bilateral cases, a team approach or staged procedures are mandatory [4,78].

Thompson et al. utilized modifications of this technique for the upper extremity. These modifications that have also been applied to the lower extremities included excising affected tissue, then creating de-epithelialized dermal flaps and folding these in toward, and suturing them to the deep investing fascia [79].

All excisional procedures produce the following advantages: (1) decrease limb size, (2) reduce episodes of cellulitis, and therefore improve the quality of life of the patients. Although these surgical procedures can be immediately effective to reduce the lymphedema volume, they can carry some risks including wound complications, swelling recurrence, and the need for the patient to wear compression garments lifelong to prevent recurrence.

##### Radical Reduction with Preservation of Perforators

RRPP consists of moderate skin excision and a subcutaneous debulking [80]. Skin markings are performed in elliptic fashion; four ellipses (two anterior and two posterior) are raised and removed after debulking the leg. The size of these ellipses of skin depends on the size of the leg and the expected amount of resection after debulking the leg. A minimum of 5 mm of skin flap are raised, preserving the subdermal plexus and subdermal fat. Both in lateral and medial aspects of the leg perforators were detected and preserved during the elevation of the medial and lateral skin flaps. Lymphedematous skin and subcutaneous tissue were then debulked off the deep fascia. Additionally, medial and lateral septae, which contain the posterior tibial and peroneal artery perforators, are preserved.

Patients affected with lymphedema (stage II and III) are considered for the RRPP procedure if the hardened subcutaneous tissue could not be excised by SAL. The final aesthetic outcome is superior to the Charles procedure; however, patients in the final stages of lymphedema with relevant skin involvement and indurated tissues continue to require the Charles procedure.

#### 4.2.3. Combined Surgical Procedures

The concept of combining physiologic and reductive procedures is set in scientific panorama for two main reasons. First, there is a lack of consensus among the experts regarding the most appropriate protocol for lymphedema treatment; indeed, each surgeon applies a surgical procedure based on his personal approach, experience and outcomes. Second, various clinical phases could co-exist in advanced lymphedema patients and the presence of a dynamic and static condition, namely a lymphatic vessel progressive failure and a lymphatic/adipose/fibrotic burden.

Excisional procedures alone are effective in reducing the lymphatic burden, the fat deposition and or the fibrotic deposition achieving extremity circumference reduction; however, they are not effective in improving the physiology of the lymphatic system, and recurrence is always a threat for patients undergoing liposuction or RRPP. Furthermore, in patients undergoing Charles’ procedure, the rate of infections and the worsening of the most distal extremity (i.e., toe) should be taken into account.

The combination with VLNT helps improve the lymphatic load of the entire extremity, especially at the most distal part of the limb (at the level of hand and foot) where excisional procedures cannot be carried out. The basis of this combined approach is that VLNT restores lymphatic fluid transit and immunocompetence, while liposuction reduces the chronic adipose deposition. Liposuction is not the only excisional procedure that can be safely combined with VLNT; indeed, RRPP and the Charles procedure were safely combined with VLNT with promising results [4,72,78,81]. From another point of view, adding excisional procedures to a physiologic approach helps reduce the lymphatic load, the rate of infection, limb volume, and improves the quality of life. The staging and the sequence of the procedures have not been standardized yet. SAL was proposed as staged before or following VLNT with satisfying results [72,82]. LVA also was staged alone or in combination with VLNT before the SAL procedure [83,84]. A one-stage two-team approach consisting of excisional procedures (Modified Charles procedure or RRPP) and concomitant VLNT has been described to address advanced lymphedema also for patients affected with bilateral extremity lymphedema; in those cases, a meticulous planning is mandatory [4,78,81]. As already reported in other fields of plastic reconstructive surgery; quality-of-life studies arose as important tools to evaluate the results based on patients’ perspective. The quality-of-life measure for limb lymphedema, commonly referred to as the acronym LYMQOL (lymphedema quality of life questionnaire), is useful in evaluating this measure and should be administered regularly. This form has questions in the four following domains: (1) symptoms; (2) body image/appearance; (3) function; and (4) mood. This tool can be useful in decision making regarding intervention and treatment, measuring responsiveness, and evaluating the cost-effectiveness of treatments [4,85,86,87].

## 5. Conclusions

Taken together, the findings of this comprehensive review highlighted that several limitations still affect the evidence supporting a specific surgical and rehabilitation treatment, albeit intense research is ongoing.

Specific strategies are needed to optimize the multidisciplinary management of this disabling condition and promote the capillarization of rehabilitation and healthy lifestyle programs.

The multidisciplinary treatment should be truly integrated for lymphedema patients, and rehabilitation should be considered the cornerstone of the multidisciplinary treatment not only for patients not suitable for surgical interventions but also before and after surgical procedures.

Therefore, an adequate management of Plastic Reconstructive Surgeons and Physical and Rehabilitation Medicine physicians should be considered as mandatory to address patients’ needs and to optimize the treatment of this disabling and detrimental condition.

## Data Availability

The datasets generated during the current study are available from the corresponding author on reasonable request.
